# Genome sequence of *Shinella* sp. strain DD12, isolated from homogenized guts of starved *Daphnia magna*

**DOI:** 10.1186/s40793-015-0129-3

**Published:** 2016-02-09

**Authors:** Anja Poehlein, Heike Freese, Rolf Daniel, Diliana D. Simeonova

**Affiliations:** Genomic and Applied Microbiology and Göttingen Genomics Laboratory, Georg-August University Göttingen, D-37077 Göttingen, Germany; Leibniz-Institut DSMZ-Deutsche Sammlung von Mikroorganismen und Zellkulturen GmbH, 38124 Braunschweig, Germany; Laboratory of Microbial Ecology, University of Konstanz, D-78457 Constance, Germany; Current Address: Laboratory of Microbial Biochemistry, Department of General Microbiology, Institute of Microbiology, Bulgarian Academy of Sciences, 26 Georgi Bonchev str., 1113 Sofia, Bulgaria

**Keywords:** *Shinella*, Phosphite assimilation, Nitrate reduction, *Alphaproteobacteria*

## Abstract

*Shinella* sp. strain DD12, a novel phosphite assimilating bacterium, has been isolated from homogenized guts of 4 days starved zooplankton *Daphnia magna*. Here we report the draft genome of this bacterium, which comprises 7,677,812 bp and 7505 predicted protein-coding genes.

**Electronic supplementary material:**

The online version of this article (doi:10.1186/s40793-015-0129-3) contains supplementary material, which is available to authorized users.

## Introduction

*Shinella* sp. strain DD12 was isolated from homogenized guts of 4 days starved zooplankton *Daphnia magna* in the frame of a study, describing the importance, diversity and stability of bacterial communities inside the *Daphnia* guts. Structural diversity of the bacterial communities were investigated over time, while *D. magna* were fed with different food sources or were let starve for 4 days, or starved to death [1, 2].

*Daphnia* spp. are small filter-feeding cladoceran zooplankton organisms which play the role of key members in the freshwater food webs. Heterotrophic bacteria can contribute significantly to the nutrition of *Daphnia* species [3, 4]. Furthermore, bacteria compared with many algae, are superior competitors for phosphorus and are often characterized by high P:C values [5]. This suggests that bacteria are a rich source of phosphorus for zooplankton [6].

Female *D. magna* were grown in water from the oligotrophic and low-phosphorus content (below 10 mg.m^-3^ concentration of total phosphorus in the water column) Lake Constance. Phosphorus in form of phosphate has been identified as the major limiting agent of phytoplankton growth in this lake [7, 8]. Studies performed in the 1990s, after a long period of active care aiming to lower the phosphorus content in the lake water, showed that the primary production of phytoplankton was not influenced substantially after the decrease of the phosphorus content [9]. This phenomenon together with the fact that some bacteria can assimilate reduced inorganic and organic phosphorus compounds (phosphite [+III] and organophosphonates) under phosphate starvation [10–19], led us to investigate the newly isolated *Shinella* sp. strain DD12 in this aspect.

The genus *Shinella* was established by An et al., in 2006, with *Shinella granuli* as type species (Ch06^T^ = JCM 13254^T^) [20, 21]. It belongs to the family *Rhizobiaceae* within *Alphaproteobacteria* and encompasses the following 6 species currently: *S. zoogloeoides**,**S. granuli**,**S. fusca**,**S. kummerowiae**,**S. daejeonensis**and**S. yambaruensis* [20–26]. The taxonomic placement of the genus *Shinella* is shown in Table 1.

**Table 1:** Classification and general features of *Shinella* sp. strain DD12

MIGS ID	Property	Term	Evidence code^a^
	Classification	Domain *Bacteria*	TAS [47]
		Phylum *Proteobacteria*	TAS [48]
		Class *Alphaproteobacteria*	TAS [49]
		Order *Rhizobiales*	TAS [21, 50]
		Family *Rhizobiaceae*	TAS [21, 51, 52]
		Genus *Shinella*	TAS [20, 53]
		Species *Shinella* sp.	TAS [20]
		Strain: DD12	TAS
	Gram stain	negative	TAS [20]
	Cell shape	Rod	IDA
	Motility	Motile	IDA
	Sporulation	Non-sporulating	NAS
	Temperature range	15–28 °C	IDA
	Optimum temperature	25 °C	IDA
	pH range; Optimum	6.6–7.5;7.0	IDA
	Carbon source	Glucose, Varied	TAS [20]
MIGS-6	Habitat	*Daphnia magna* gut	IDA
MIGS-6.3	Salinity	0.5–5 % NaCl	IDA
MIGS-22	Oxygen requirement	Aerobic	TAS [20]
MIGS-15	Biotic relationship	free-living/host/commensal	IDA
MIGS-14	Pathogenicity	non-pathogen	NAS
MIGS-4	Geographic location	Germany/Constance	IDA
MIGS-5	Sample collection	November 2008	IDA
MIGS-4.1	Latitude	47.689081	IDA
MIGS-4.2	Longitude	9.187099	IDA
MIGS-4.4	Altitude	405 m; a.s.l.	IDA

^a^Evidence codes - IDA: Inferred from Direct Assay; TAS: Traceable Author Statement (i.e., a direct report exists in the literature); NAS: Non-traceable Author Statement (i.e., not directly observed for the living, isolated sample, but based on a generally accepted property for the species, or anecdotal evidence). These evidence codes are from the Gene Ontology project [54]

*Shinella* sp. strain DD12 was chosen for sequencing as it is able to assimilate phosphite under phosphate starvation and use it as single P- source to support its growth. We also focus on the following specific features of this genome - the assimilation of inorganic and organic phosphonates, providing that the organophosphonates are known to serve not only as P-, but as C- and N-sources for different bacteria. This is the first report on a genome sequence of a member of genus *Shinella*.

## Organism information

### Classification and features

*Shinella* sp. DD12 is an aerobic, motile, Gram-negative, non-spore-forming, rod-shaped, hemoheterotroph and psychrotolerant bacterium.

**Fig. 1:**
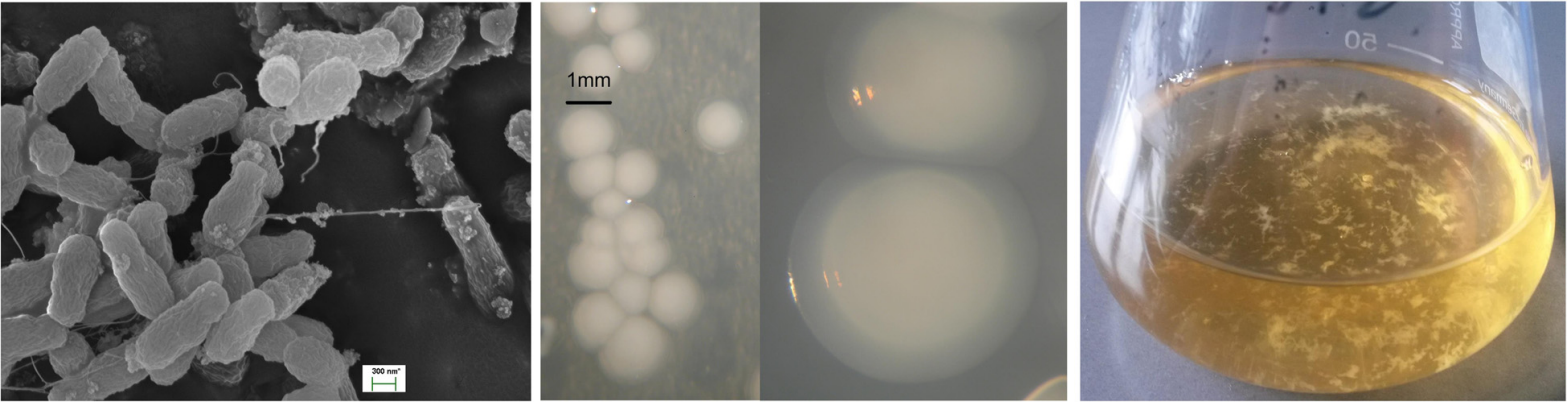
Images of *Shinella* sp. strain DD12 using scanning (*left*) electron microscopy and the appearance of colony morphology on solid (*middle*), and liquid (*right*) nutrient agar medium

The cells of strain DD12 are short rounded rods with blunt ends and size of 0.6–1 μm in length, and 0.3–0.5 μm in width. Cells are motile via monotrichous flagellum (Fig. 1, Left).

*Shinella* sp. DD12 forms colonies within 3 to 5 days, when grown on nutrient agar at 18 °C (Fig. 1, Center). Colonies are circular, raised to convex, smooth milky-white in color, slightly opaque with pronounced translucent halo-like edges. In liquid media cells form white fluffy aggregates with finger-like or tree-like morphology (Fig. 1, Right). The strain grows at the temperature range of 10–30 °C. No growth was observed at 37 °C. At 18 °C the strain grows poorly on nutrient broth. At 21 °C it grows with a doubling time of 54–61 h on nutrient broth. By employing a newly developed chemically defined medium (MDS3) with phosphate as the phosphorus source the doubling time was reduced to 32–33 h at 21 °C. The composition of MDS3 medium and the conditions of the tests for phosphite assimilation are available in Additional file 1.

*Shinella* sp. strain DD12 is positive for catalase, catalase-peroxidase, β-galactosidase and β-glucosidase activity as described for all members of the genus [20, 23]. Strain DD12 can grow oxidatively with the production of acid on different sugars and sugar alcohols. *Shinella* sp. strain DD12, like other *Shinella* species except *S. fusca**,* cannot grow on melibiose or starch [20, 23–26]. It does not either grow on inulin as is found for *S. kummerowiae*, whereas there is no data reported for the rest of *Shinella* strains. Strain DD12 however, shows some specificity in substrate assimilation, as the lack of growth on D-arabinose, while all *Shinella* strains can grow on this substrate with exception of *S. yambaruensis* [20, 26]. Analogously, a weak growth on salicin was observed for *Shinella* sp. strain DD12, where five of the six *Shinella* strains cannot grow on this substrate. *S. granuli* growth on salicin remains undetermined [20, 25].

**Fig. 2:**
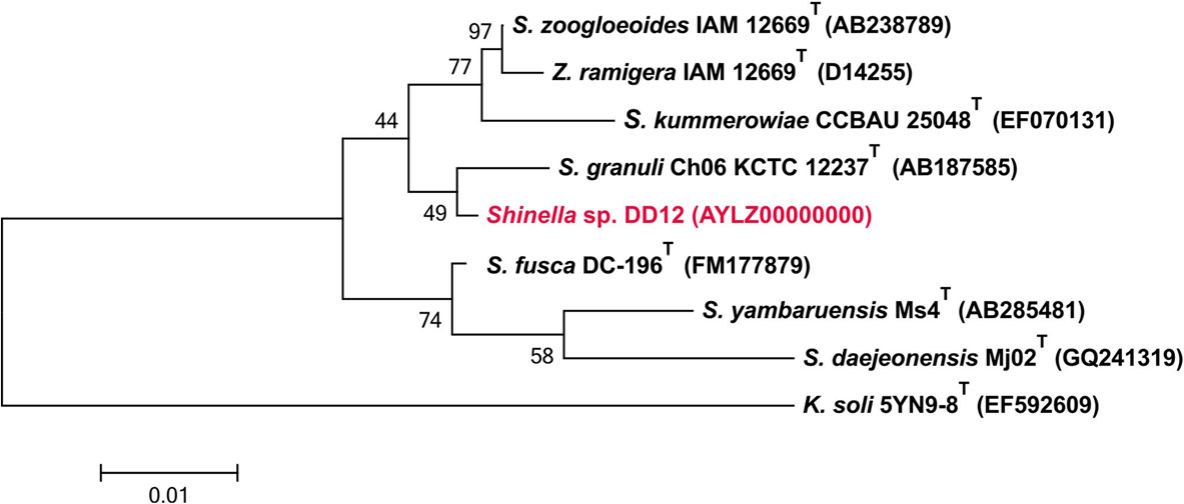
Phylogenetic tree highlighting the position of *Shinella* sp. strain DD12, based on 16S rRNA gene sequences

We compared 16S gene sequences of *Shinella* sp. DD12 with the non-redundant nucleotide collection of NCBI using NCBI MegaBLAST [27, 28]. This comparison revealed that the strain shares 99 % (1445/1453 bp) and 99 % (1438/1446 bp) sequence identity to the 16S rRNA gene sequences of *Rhizobium* sp. R-24658, and *S. zoogloeoides* 81 g, respectively. Figure 2 shows the phylogenetic neighborhood of *Shinella* sp. DD12 in a 16S rRNA sequence based tree of all *Shinella* type species.

The phylogenetic tree was calculated with MEGA5 [29] using the Maximum Likelihood method based on the Jukes Cantor model [30]. Sequences were downloaded from the RDP [31], aligned by CLUSTALW [32] and tested by the bootstrap approach with 1000 resamplings. The length of the tree branches was scaled according the number of substitutions per site (see size bar). *Shinella* sp. DD12 clustered together with *S.granuli* Ch06T KCTC12237.

The minimum information about the genome sequence (MIGS) is provided in Table 1, according to MIGS recommendations [33].

## Genome sequencing information

### Genome project history

This bacterium was selected for sequencing on the basis of its environmental relevance to issues in global P- and N-cycles, and still widely unrecognized reduced P-cycle in nature. Prior to sequencing, *Shinella* sp. strain DD12 was tested for growth in a newly developed chemically defined liquid medium MDS3 supplemented with 1 mM sodium phosphite as single P-source. The growth and the phosphite assimilation ability of this isolate were confirmed at physiological level (three successive passages in triplicate). The genome project has been deposited in GenBank database (AYLZ00000000) and as an improved high-quality-draft genome sequence in IMG. Genome Sequencing and annotation were done at Göttingen Genomics Laboratory; while cultivation and analysis were performed at the University of Konstanz. The project information and its association with MIGS version 2.0 compliance [33] are presented in Table 2.

**Table 2:** Project information

MIGS ID	Property	Term
MIGS 31	Finishing quality	Improved high-quality draft
MIGS-28	Libraries used	Illumina paired-end library (1 kb insert size)
MIGS 29	Sequencing platforms	Illumina GAII
MIGS 31.2	Fold coverage	75 × Illumina
MIGS 30	Assemblers	SPAdes 2.5
MIGS 32	Gene calling method	YACOP, Glimmer
	Locus Tag	SHLA
	Genbank ID	AYLZ00000000
	GenBank Date of Release	2014-07-15
	GOLD ID	Gp0043937
	NCBI project ID	223517
	BIOPROJECT	PRJNA223517
MIGS 13	Source Material Identifier	DD12
	Project relevance	Ecology, Biotechnology

### Growth conditions and genomic DNA preparation

*Shinella* sp. DD12 was grown either in nutrient broth or on nutrient agar. The medium was adjusted to pH 7.0 and autoclaved for 25 min at 125 °C. MDS3 medium was used to assay carbohydrate, phosphite and phosphate assimilation by the strain. The chemical composition of the MDS3 medium is given in Additional file 1.

The genomic DNA of the strain was isolated as follows: the cells from 4 ml of a well grown culture in nutrient broth reaching an OD_600_ of 0.348 ± 0.050 were harvested at 13,000 × *g* in a benchtop microfuge for 5 min. Cell pellet was suspended in the cell lysis solution of the Purgene Core Kit B (Qiagen, Hilden, Germany). Further, the genomic DNA extraction processed as recommended by the manufacturer. DNA quantity was determined with NanoDrop ND-1000 to ensure that the concentration is greater than 30 ng/μl. One nanogram of the genomic DNA was used for sequencing.

### Genome sequencing and assembly

Extracted DNA was used to prepare shotgun libraries for the Genome Analyzer II (Illumina, San Diego, CA, USA). Libraries were prepared according to the manufacturer protocol. Sequencing resulted in 7,118,226 paired-ends Illumina reads of 112 bp and a 72.54-fold coverage. Reads were trimmed using Trimmomatic 0.32 [34] to remove sequences with quality scores lower than 20 (Illumina 1.9 encoding) and remaining adaptor sequences. The initial hybrid *de novo* assembly employing the SPAdes 2.5 [35] software resulted in 236 contigs larger than 0.5 kb of which 162 were larger than 1 kb including 139 contigs larger than 3 kb. The final assembly had an N50 value of 97,231 bp and an N90 value of 24,331 bp.

### Genome annotation

YACOB and GLIMMER [36] software tools were used for automatic gene prediction. RNAmmer [37] and tRNAscan [38] were used for identification of rRNA and tRNA genes, respectively. Functional annotation of the predicted protein-coding genes was carried out with the IMG/ER system [39] and was manually curated by using the Swiss-Prot, TrEMBL, and InterPro databases [40].

## Genome properties

The genome statistics are provided in Table 3. The pseudogenes may also be counted as protein coding or RNA genes, so they are not additive under total gene count.

**Table 3:** Genome statistics

Attribute	Value	% of total
Genome size (bp)	7,677,812	100.00
DNA coding (bp)	6,843,125	89.13
DNA G + C (bp)	4,867,601	63.40
DNA scaffolds	235	100.00
Total genes	7555	100.00
Protein coding genes	7505	99.34
RNA genes	50	0.66
Pseudo genes	164	2.17
Genes in internal clusters	6245	82.66
Genes with function prediction	6241	82.64
Genes assigned to COGs	5394	71.40
Genes with Pfam domains	6268	82.96
Genes with signal peptides	681	9.01
Genes with transmembrane helices	1725	22.83
CRISPR repeats	0	0

The draft genome of *Shinella* sp. DD12 consists of 236 contigs comprising 7.678 Mb and an overall GC content of 63.40 mol%. The genome harbors 7555 putative genes, of which 7505 are protein-encoding and 50 RNAs (2 rRNA and 48 tRNA). The tRNAs included tRNA necessary for selenocystein incorporation (SHLA_2c001070). Protein encoding genes with a putative function prediction are 6241 (82.61 %) of all proteins in the genome and 1264 (16.73 %) were annotated as hypothetical proteins. The majority of the protein-encoding genes 5394 (71.40 %) were assigned to one of the known COG categories [41]. The distribution of these genes with respect to assigned functions is presented in Table 4.

**Table 4:** Number of genes associated with general COG functional categories

Code	Value	% age	Description
J	206	3.07	Translation, ribosomal structure and biogenesis
A	0	0.00	RNA processing and modification
K	666	9.92	Transcription
L	259	3.86	Replication, recombination and repair
B	7	0.10	Chromatin structure and dynamics
D	52	0.77	Cell cycle control, Cell division, chromosome partitioning
V	65	0.97	Defense mechanisms
T	334	4.97	Signal transduction mechanisms
M	261	3.89	Cell wall/membrane biogenesis
N	88	1.31	Cell motility
U	111	1.65	Intracellular trafficking and secretion
O	177	2.64	Posttranslational modification, protein turnover, chaperones
C	338	5.03	Energy production and conversion
G	605	9.01	Carbohydrate transport and metabolism
E	955	14.22	Amino acid transport and metabolism
F	130	1.94	Nucleotide transport and metabolism
H	226	3.37	Coenzyme transport and metabolism
I	218	3.25	Lipid transport and metabolism
P	407	6.06	Inorganic ion transport and metabolism
Q	194	2.89	Secondary metabolites biosynthesis, transport and catabolism
R	781	11.63	General function prediction only
S	634	9.44	Function unknown
-	1535	20.32	Not in COGs

The total is based on the total number of protein coding genes in the genome

## Insights from the genome sequence

The genome of *Shinella* sp. DD12 consists of a circular chromosome and at least 7 plasmids as we could detect 7 different *repABC* gene clusters located on 7 different contigs. Further database analysis revealed that all complete sequenced *Rhizobiaceae* genomes harbor usually between 2 and 6 plasmids, but species with up to 9 plasmids have been found, as in *Ensifer fredii* HH103 [42].

The strain is aerobe and its aerobic respiratory chain contains all genes encoding Complex I to Complex V. In addition, strain DD12 possesses a complete denitrification pathway via periplasmic cytochrome *c* [43]. The pathway found in this genome includes the genes encoding a periplasmic nitrate reductase *napABC* (SHLA_29c000730 - SHLA_29c000770), NO-forming nitrtite reductase *nirK* (SHLA_5c000410), nitric oxide reductase *norCBD* (SHLA_5c000290 - SHLA_5c000340) and a nitrous oxide reductase *nosZ* (SHLA_36c000580). The genome analysis of the strain DD12 revealed the potential abilities of this isolate to reduce nitrogen via the dissimilatory nitrate reduction to ammonia (DNRA) pathway, and to assimilate nitrate to L-glutamine and L-glutamate. The genes encoding nitrogen fixation ability such as N-acetylglucosaminyl transferase (*nodC*) or nitrogenase reductase (*nifH*) are absent from the genome. This is consistant with the previously reported lack of nitrogen fixation ability in free-living *Shinella* species, except for the only known symbiont *S. kummerowiae* [20, 23, 25].

**Fig. 3:**
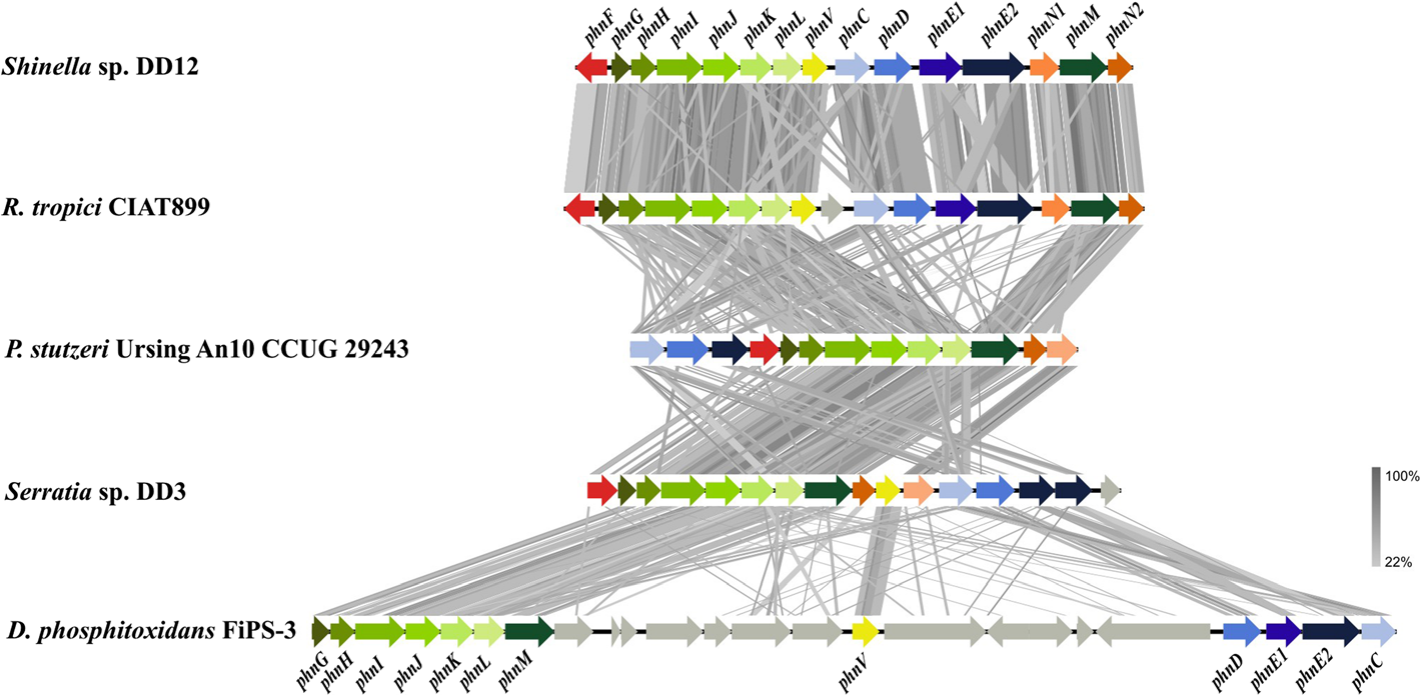
Tblastx comparison of the C-P lyase complex. An E-value cutoff of 1e^-10^ was used and visualization was done with the program Easyfig [55]. Functional genes of the C-P lyase complex were marked in *green*, accessory genes in *orange*, the ABC-type transporter in *blue* and the regulatory subunit in *red*. Genes not directly associated with this pathway are marked in *grey*

*Shinella* sp. strain DD12 is able to utilize reduced inorganic phosphonate (phosphite) and presumably organophosphonates as the single P-sources to support its growth. The phosphite oxidation most probably proceeds through a periplasmic alkaline phosphatase (*phoA*), analogously to *E.coli* [18], or through the carbon-phosphorus (C-P) lyase complex [10–12, 15]. The latter complex is known to have broader substrate specificity, including the oxidation of phosphite and the assimilation of the most common organophosphonate - methylphosphonate. The C-P lyase complex, although the presence of the conserved structural *phnGHIJKLM* gene cluster, shows low conservation level of the gene sequences arrangement amongst representatives of *Alpha*- *Beta*- *Gamma*- and *Deltaproteobacteria* (Fig. 3). However, this drastically changes within the *Rhizobiaceae* members harboring a C-P lyase complex. The C-P complex shows highest conservation level of the gene sequences and their arrangement amongst the *Rhizobiaceae* members that harbors it.

**Fig. 4:**
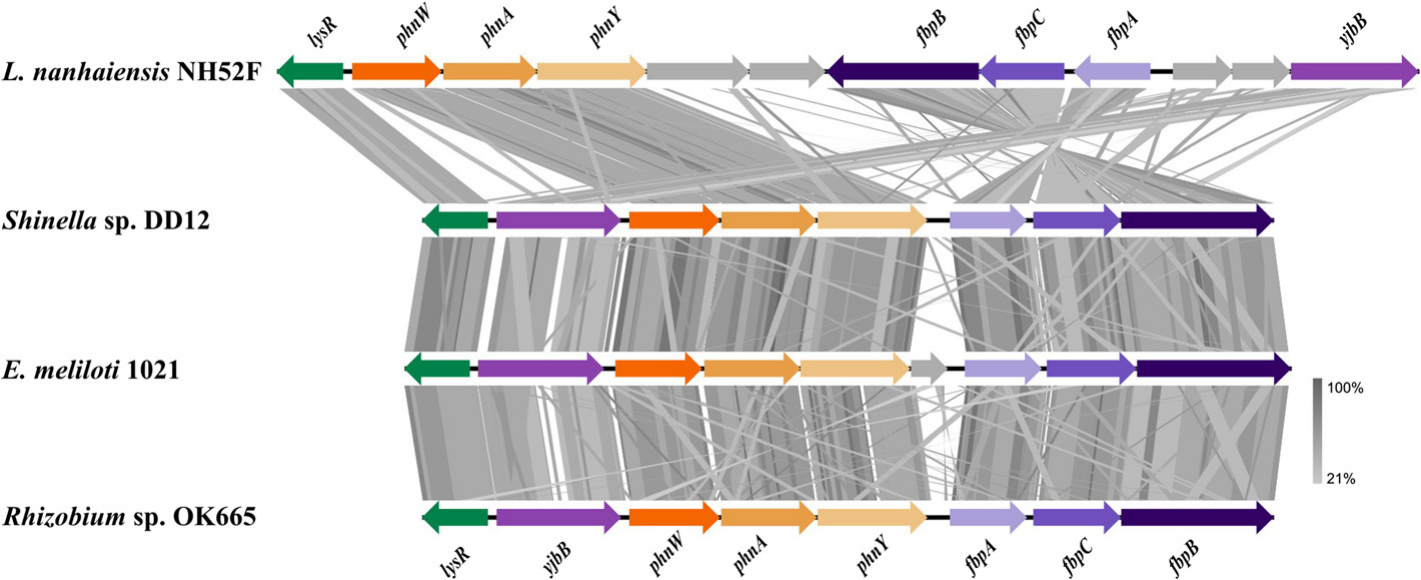
Tblastx comparison of *Alphaproteobacteria phnWAY* operon. An E-value cutoff of 1e^-10^ was used and visualization was done with the program Easyfig [55]. The *phnWAY* cluster was marked in *orange* tones; sodium/phosphonate symporter (*yjbB*) was marked in *purple*; the genes coding ABC-type ferric uptake system (*fbpABC*) were marked in *blue*; the transcriptional regulator (*lysR*) is shown in *green*

In addition, the *Shinella* sp. DD12 genome harbors a 2-aminoethylphosphonate (2-AEP) degradation pathway, which operates through the phosphonoacetaldehyde dehydrogenase - phosphonoacetate hydrolase (*phnWAY*) [16, 44]. The 2-AEP (ciliatine) is a common phosphonate constituent of the phospholipids in a variety of marine invertebrates, including ciliated protozoa, sees anemones, some plants and animals. Recently, the synthesis of sphingophosphonolipids was found in some bacterial species including *Bacteriovorax stolpii*, a facultative predator which parasitizes larger Gram-negative bacteria [45]. A Tblastx comparison of the *phnWAY* encoding operon from *Shinella* sp. strain DD12 with another 3 species belonging to *Alphaproteobacteria*, two of which members of *Rhizobiaceae* is shown on Fig. 4. An analysis of all genomes available at IMG (as of April 1, 2015) against phosphonoacetaldehyde dehydrogenase encoding gene (*phnY*) revealed its presence in 431 gene clusters. However, the complete *phnWAY* operon was present in only 92 genomes of which 41 belong to *Rhizobiaceae* species. Furthermore, the *phnWAY* operon was placed in the majority of the *Alphaproteobacteria* genomes in close proximity to the *fbpABC* transporter involved in the utilization of xenosiderophores as iron sources in a TonB-independent manner. It is known that the *fbpABC* gene cluster is transcribed as separate operon in *Neisseria meningitidis* [46]. However, whether this cluster plays a role in phosphonate uptake in the cell is unclear.

## Conclusions

The draft genome sequence of *Shinella* sp. strain DD12 described here is the first genome sequence of a member of the genus *Shinella*. The genome of the strain DD12 suggests the presence of 7 plasmids, which is often found amongst members of *Rhizobiaceae*.

The genome analysis of *Shinella* sp. strain DD12 indicates that the bacterium is a denitrifier, as it harbours two complete sets of genes encoding: i) the dissimilatory nitrate reduction to ammonia pathway and ii) assimilative nitrate reduction to L-glutamine, and L-glutamate pathway. *Shinella* sp. strain DD12 cannot fix nitrogen, similarly to the other free-living known *Shinella* species, whereas the symbiotically growing *S. kummerowiae* is a nitrogen fixing bacterium.

Finally, the genome of *Shinella* sp. DD12 encodes three complete pathways for assimilation of phosphonates. The presence of these three pathways indicates relatively broad abilities to utilise reduced phosphonates as P- and/or C- and N-sources, compared to the remaining genomes of *Rhizobiaceae* members and even to *Alphaproteobacteria* as a whole. This could be a great advantage for the strain DD12 in environments where other bacteria can face growth limitations, providing that the inorganic- and organophosphonates are naturally occurring compounds. Furthermore, the presence of the genes encoding the complete pathway for 2-AEP containing biomolecules might provide a defence mechanism against predator and parasite bacteria.

## Additional file

Additional file 1:
**Composition of MDS3 medium and growth conditions in phosphite assimilation tests.** (PDF 64.3 kb)

## Abbreviations

RDP: Ribosomal Database Project (East Lansing, MI, USA)
