# Supplementary material for: Genome sequence of Shinella sp. strain DD12, isolated from homogenized guts of starved Daphnia magna
Source: Stand Genomic Sci. 2016 Feb 9;11:14. doi: 10.1186/s40793-015-0129-3 (PMC4748535; doi:10.1186/s40793-015-0129-3)
Supplement: Additional file 1: — Composition of MDS3 medium and growth conditions in phosphite assimilation tests. (PDF 64.3 kb) [file 40793_2015_129_MOESM1_ESM.pdf]

Supplementary Data S1:

Composition of MDS3 medium

The chemically defined medium MDS3, developed to study phosphite and phosphate uptake and assimilation was developed on the basis of the medium described initially for the isolation of *Pseudomonads* from Stanier and co-authors in 1966 [1]. This medium had undergone several modifications, where the most popular one was that from Palleroni and co-authors [2]. A modified Palleroni's medium was used from Freese during the isolation of bacterial strains from the guts of *D. magna* (unpublished). Further the medium has been adjusted for the needs of the phosphite/phosphonate assimilation assays, by Simeonova [3].

<u>Ingredients and preparation:</u>

20mM Tris-HCl, pH: 7.0-7.2; Alternatively, MOPS or HEPES buffer can be used as well.

<u>Solution 1 (in g.l^{-1}):</u> $MgSO_4.7H_20$ - 0.12; NH_4Cl - 0.27; KCl - 0.5; NaCl - 1.0; prepared as 10x stock solution and autoclaved at 121°C for 25 min;

<u>Solution 2:</u> $CaCl_2.6H_2O$ - 0.132g in 10 ml ddH_2O, autoclaved at 121°C for 25 min;

<u>SL10 Solution [4]</u>: Trace Elements Solution 10;

<u>7 Viamine Solution [5]</u> - filter sterilized;

<u>MDS3 preparation (1l):</u> Tris-HCl buffer (10x stock) – 100 ml;

Solution 1 (10x stock) – 100 ml;

Solution 2 - 0,1 ml;

Trace Elements Solution 10 - 1 ml;

7 Vitamins Solution 10 - 1 ml;

q.s.p. with autoclaved doubly distilled H_2O to 1l.

As a single phosphorus source 0.1 to 1mM phosphite or phosphate were supplemented into the MDS3. Glucose 10mM was used as a carbon source.

The medium is suitable for analogous studies with organophosphonates, since it does not include any phosphorus containing chemicals.

References:

1. Stanier R., Palleroni N., Doudoroff M.: **The aerobic pseudomonads: a taxonomic study.** J Gen Microbiol 1966, **43**(2):159-271.
2. Palleroni N.J., Doudoroff M., Stanier R.Y., Solanes R.E., Mandel M.: **Taxonomy of the aerobic *Pseudomonads*: the properties of the *Pseudomonas stutzeri* group**. Microbiology 1970, **60**(2):215-231. http://mic.microbiologyresearch.org/content/journal/micro/10.1099/00221287-60-2-215
3. Poehlein A., Daniel R., Simeonova D.D.: **Genome sequence of Pedobacter glucosidilyticus DD6b, isolated from zooplankton Daphnia magna**. Standards in Genomic Sciences 2015, **10**(1):1-9. http://dx.doi.org/10.1186/s40793-015-0086-x
4. Widdel F., Kohring G.-W., Mayer F.: **Studies on dissimilatory sulfate-reducing bacteria that decompose fatty acids**. Arch Microbiol 1983, **134**(4):286-294. http://dx.doi.org/10.1007/BF00407804
5. Widdel F., Pfennig N.: **Studies on dissimilatory sulfate-reducing bacteria that decompose fatty acids. I. Isolation of a new sulfate-reducer enriched with acetate from saline environments-description of *Desulfobacter postgatei* gen. nov. sp. nov.** Arch Microbiol 1981, **129**:395-400. http://dx.doi.org/10.1007/BF00406470
